# Research Advances in Nanosensor for Pesticide Detection in Agricultural Products

**DOI:** 10.3390/nano15141132

**Published:** 2025-07-21

**Authors:** Li Feng, Xiaofei Yue, Junhao Li, Fangyao Zhao, Xiaoping Yu, Ke Yang

**Affiliations:** 1 College of Agriculture, Forestry and Medicine, The Open University of China, Beijing 100039, China; fengli@ouchn.edu.cn; 2 Rehabilitation Pharmacy Center, Affiliated Beijing Rehabilitation Hospital, Capital Medical University, Beijing 100144, China; yuexiaofei@mail.ccmu.edu.cn; 3 School of Life Sciences, China Jiliang University, Hangzhou 310018, China; p24091055031@cjlu.edu.cn (J.L.); p24091055084@cjlu.edu.cn (F.Z.); 4 State Key Laboratory for Quality Ensurance and Sustainable Use of Dao-di Herbs, Beijing 100700, China

**Keywords:** nanosensors, agricultural products, pesticide residue detection

## Abstract

Over the past few decades, pesticide application has increased significantly, driven by population growth and associated urbanization. To date, pesticide use remains crucial for sustaining global food security by enhancing crop yields and preserving quality. However, extensive pesticide application raises serious environmental and health concerns worldwide due to its chemical persistence and high toxicity to organisms, including humans. Therefore, there is an urgent need to develop rapid and reliable analytical procedures for the quantification of trace pesticide residues to support public health management. Traditional methods, such as chromatography-based detection techniques, cannot simultaneously achieve high sensitivity, selectivity, cost-effectiveness, and portability, which limits their practical application. Nanomaterial-based sensing techniques are increasingly being adopted due to their rapid, efficient, user-friendly, and on-site detection capabilities. In this review, we summarize recent advances and emerging trends in commonly used nanosensing technologies, such as optical and electrochemical sensing, with a focus on recognition elements including enzymes, antibodies, aptamers, and molecularly imprinted polymers (MIPs). We discuss the types of nanomaterials used, preparation methods, performance, characteristics, advantages and limitations, and applications of these nanosensors in detecting pesticide residues in agricultural products. Furthermore, we highlight current challenges, ongoing efforts, and future directions in the development of pesticide detection nanosensors.

## 1. Introduction

**Figure 1:**
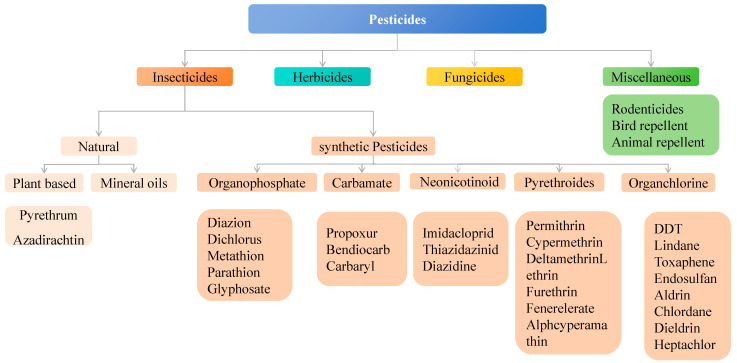
Different types of pesticides used in agriculture [3,4,5]. Adapted with permission from ref. [3]. Copyright 2019, ExcellentPublishers; Adapted with permission from ref. [4]. Copyright 2020, Elsevier; Adapted with permission from ref. [5]. Copyright 2016, Elsevier.

According to the definition provided by the Food and Agriculture Organization of the United Nations, pesticides are chemical substances, biological components, or mixtures of both that can be used to control pests and microorganisms, eliminate weeds, or regulate plant growth [1]. Over the past few decades, hundreds of pesticides have been developed and widely applied in agriculture (Figure 1). Among them, organochlorine pesticides exhibit high toxicity to many organisms and are poorly biodegradable. Some compounds can persist in the environment for more than 30 years. Therefore, the use of organochlorine pesticides has been banned in many countries and replaced by more rapidly biodegradable alternatives, such as carbamates and neonicotinoids [2].

Pesticides pose serious threats to both the environment and human health by contaminating soil and groundwater, disrupting biodiversity, and accumulating along the food chain. They have been linked to various diseases, including cancer, asthma, allergic reactions, chronic illnesses, and neurological disorders [3,6] (Table 1).

Pesticides comprise a wide variety of chemical structures and classifications, leading to significant differences in their physicochemical properties. In addition, their residue levels are usually found at trace concentrations, necessitating the development of highly sensitive, selective, and rapid analytical methods that are also user-friendly for reliable detection [7]. In recent years, considerable scientific attention has been devoted to advancing pesticide detection methodologies, resulting in the development of diverse analytical techniques such as capillary electrophoresis (CE), ultraviolet–visible (UV-Vis) spectroscopy, Fourier-transform infrared (FTIR) spectroscopy, nuclear magnetic resonance (NMR) spectroscopy, and chromatographic methods including high-performance liquid chromatography (HPLC) and gas chromatography (GC).

While conventional pesticide detection techniques (e.g., GC, HPLC) offer satisfactory sensitivity and throughput, they are constrained by their reliance on specialized costly laboratory equipment and trained personnel. Additionally, these methods are time-consuming, involve tedious sample preparation, and are unsuitable for rapid, on-site, or real-time analysis [8]. These limitations underscore an urgent need for innovative detection platforms that integrate analytical sensitivity, methodological robustness, and field-deployable simplicity. Recent rapid advancements in sensing technology provide promising solutions to address these challenges.

**Table 1:** The main harmfulness and poisoning mechanism of the representative categories of pesticides to human health.

Categories	Representing Pesticides	Poisoning Mechanism	Harmfulness	Ref.
Organopsphorus	Parathion, systox, malathion, dimethoate, dichlorvos, dichlorvos	Irreversible inhibiting of AChE	Dyspnea, pulmonary edema, muscle spasms, renal dysfunction, dizziness, headache, palpitations, chest tightness, bradycardia	[9]
Carbamate	Carbaryl, metolcarb, aldicarb, carbofuran	Similar to organopsphorus, but with higher reversibility	Dizziness, fatigue, blurred vision, nausea and vomiting, abdominal pain, excessive sweating, muscle fiber tremors, breathing difficulties, coma, liver and kidney function damage	[10]
Neonictinoids	Imidacloprid, thiamethoxam, imidacloprid, fipronil	Continuously activating nicotinic ACh receptors	Nausea, vomiting, headache, dizziness, insomnia, anxiety, consciousness disorders	[11]
Pyrethroides	Permethrin, deltamethrin, fenvalerate, cyfluthrin	Induce neurotoxicity by interfering with sodium channels and GABA receptors	Skin rashes, nausea, vomiting, abdominal pain, diarrhea, headache, dizziness, anxiety, confusion, cough, endocrine disorders	[12]
Organchlorine	Hexachlorocyclohexane, indene octachloride, toxaphene, indene heptachloride	Inhibiting GABA receptors, generating ROS, disrupting inositol metabolism and endocrine system	Skin rashes, nausea, vomiting, abdominal pain, diarrhea, headache, dizziness, anxiety, confusion, cough, endocrine disorders	[13]

**Figure 2:**
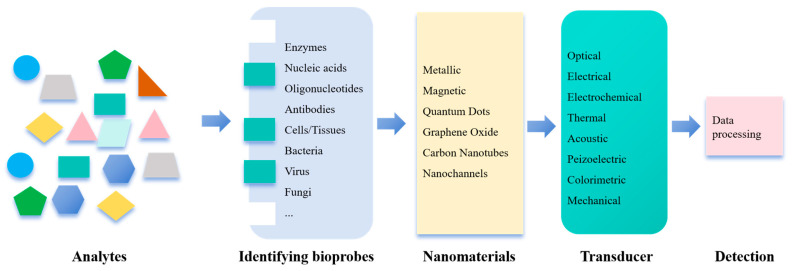
Schematic representation of major components of sensors.

A sensor is a device that converts the presence or concentration of target analytes into quantifiable electrical or optical signals through the coordinated actions of recognition elements (receptors), signal transducers, and output modules (detectors) (Figure 2) [14,15,16].

A nanosensor, engineered using materials in the nanoscale range (0.1–100 nm), leverages unique physicochemical properties and exhibits superior performance compared to traditional sensors, thereby helping to overcome the limitations of conventional pesticide residue detection techniques [17,18]. Nanosensors have the following merits: (1) user-friendly operation with enhanced sensitivity and specificity, enabling on-site and real-time detection; (2) rapid and selective recognition capabilities in complex matrices (e.g., milk, vegetables, and fruits); and (3) simplified structural designs that enable cost-effective fabrication (e.g., screen-printed electrodes, test strips) [19].

This review article summarizes recent advancements in various types of pesticide-detecting nanosensors, with an emphasis on the use of enzymes, antibodies, aptamers, and molecularly imprinted polymers (MIPs) as recognition elements. In this context, the applications of these receptors in detection strategies—primarily involving electrochemical and optical sensing—for monitoring pesticide residues in agricultural products are comprehensively discussed. Furthermore, the nanomaterials used, working principles, advantages, and limitations of different nanosensor platforms are critically evaluated. In preparing this review, we primarily searched the PubMed database for the literature published in the past 5 years (2015–2025) using the keywords “nanosensors,” “pesticide detection,” and “agricultural products.” Due to the rapid expansion of research in this field, we sincerely apologize to any authors whose valuable contributions may have been inadvertently overlooked.

## 2. Common Recognition Elements for Pesticide Detection

A recognition element (receptor) is a critical component of a sensor, designed to interact selectively with the specific analyte of interest and generate a measurable signal through the transducer. High selectivity toward the target analyte in complex matrices is a key requirement for the receptor. Various types of receptors, such as antibodies, enzymes, nucleic acid probes (including aptamers), cellular structures, and biomimetic materials, can be employed in sensor design. Among these, enzymes, antibodies, aptamers, and molecularly imprinted polymers (MIPs) have been most widely integrated into different types of sensors for pesticide detection. This section focuses on the key characteristics of these receptors and their applications, primarily in optical and electrochemical sensors for pesticide monitoring.

### 2.1. Enzyme

Enzymes are biological catalysts produced by living cells, most of which are proteins, with a small subset consisting of catalytic RNA molecules. Enzyme-based biosensors utilize enzymes as biorecognition elements to convert biochemical reactions into measurable optical or electrochemical signals. Due to their simplicity and compatibility with field-deployable formats, enzyme-based sensors dominate pesticide residue analysis.

The advantages of enzyme probes include high selectivity, sensitivity, rapid response, operational simplicity, automation compatibility, and relatively low maintenance costs. However, enzymes also exhibit notable drawbacks, including limited stability and shelf life, sensitivity to environmental factors, complex and costly preparation, risk of fluctuating sensitivity, and potential cross-reactivity. Enzyme stability is particularly critical, and emerging solutions include the development of nanoenzymes with enhanced substrate specificity and enzyme-mimicking MIPs.

**Figure 3:**
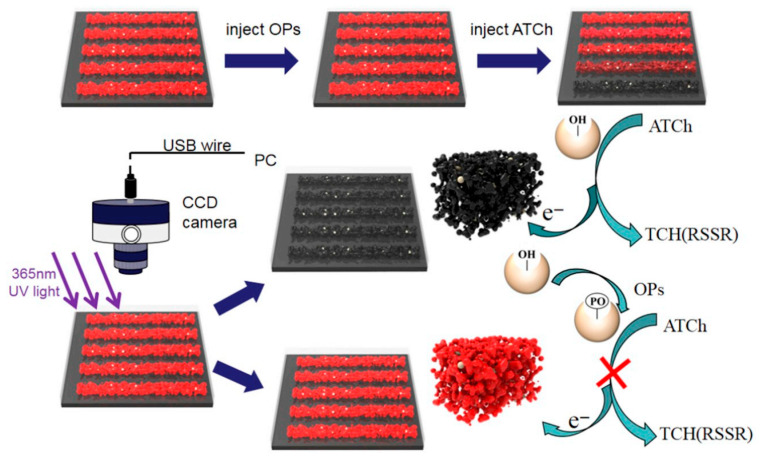
Schematic illustration of visual detection of OPs based on enzyme inhibition recovering the fluorescence of CdTe aerogel [20]. Adapted with permission from ref. [20]. Copyright 2019, Elsevier.

Most enzyme-based sensors rely on enzyme inhibition mechanisms (e.g., inhibition of acetylcholinesterase (AChE) by organophosphorus pesticides, OPs). Hu et al. [20] developed a fluorescent microfluidic sensor for OP detection by integrating a 3D CdTe quantum dot (QD) aerogel with microfluidic technology. This sensor operates through fluorescence quenching: the red fluorescence of the QD aerogel is quenched by thiocholine (produced via AChE-catalyzed hydrolysis of acetylthiocholine, ATCh) due to photoinduced electron–hole pair dissociation in QDs. In the presence of OPs, AChE activity is inhibited, preventing ATCh hydrolysis and restoring QD fluorescence intensity. This visually detectable system achieved a detection limit (LOD) of 0.38 pM for OPs in apples and could quantify total OP content in mixed samples with unknown pesticide types (Figure 3).

Nanoenzymes, which are nanomaterials with enzyme-mimicking catalytic activity, have emerged to overcome limitations associated with natural enzymes. Arsawiset and coworkers [21] designed a paper-based analytical device using copper oxide nanoparticles (CuONPs) as nanoenzymes. These CuONPs exhibit peroxidase-like activity, catalyzing the oxidation of o-dianisidine in the presence of hydrogen peroxide (H_2_O_2_) produced from ATCh hydrolysis. Exposure to OPs inhibits AChE, reducing color intensity, which is quantifiable via smartphone imaging. The device showed a good linear detection range (0.1–5 mg/L), an LOD of 0.08 mg/L, and a short analysis time of ~10 min for malathion detection. It was successfully applied to fruits and vegetables with high accuracy and portability.

**Figure 4:**
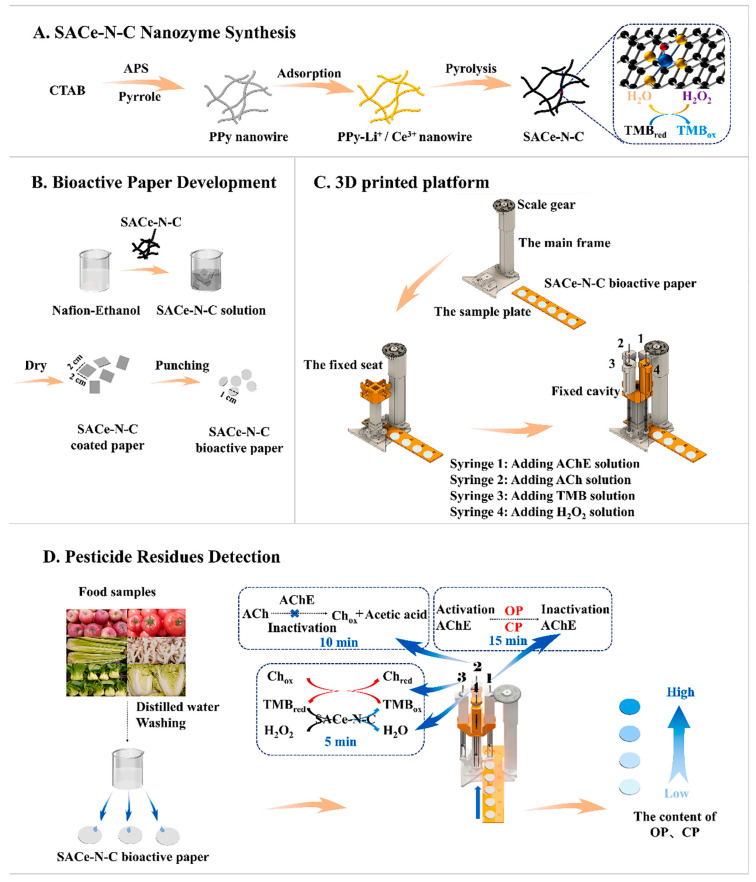
Principle and process of detecting organophosphorus carbamate pesticide residues with SACe-N-C nanozyme bioactive paper (PPy, nanowire polypyrrole; CTAB, cetyltrimethyl ammonium bromide; APS, ammonium peroxydisulfate; OP, organophosphorus pesticides; CP, carbamate pesticides; AChE, acetylcholinesterase; Ach, acetylcholine; Ch, choline) [22]. Adapted with permission from ref. [22]. Copyright 2022, Elsevier.

While enzyme-mimicking materials show promise, unstable catalytic activity remains a barrier. Recently, single-atom nanozymes (SAzymes) have shown potential, offering enhanced stability, electronic modulation, and higher turnover frequencies by presenting isolated active sites. Song et al. [22] developed an SAzyme (SACe-N-C) exhibiting peroxidase-like activity and combined it with AChE in a colorimetric biosensor. The system operates via a cascade mechanism: SACe-N-C catalyzes H_2_O_2_ decomposition, producing hydroxyl radicals (·OH) that oxidize 3,3′,5,5′-tetramethylbenzidine (TMB) to a blue oxidized form. OPs inhibit AChE, decreasing choline production, which, in turn, reduces TMB oxidation. This paper-based platform, coupled with a 3D-printed detection system, achieved LODs of 55.83, 71.51, 81.81, and 74.98 ng/mL for omethoate, methamidophos, carbofuran, and carbosulfan, respectively, enabling on-site analysis within 30 min (Figure 4).

Nano-ELISA technologies integrate enzyme-linked immunoassays with nanomaterials, significantly enhancing sensitivity [23]. Chen et al. [23] engineered 3D protein-inorganic hybrid nanoflowers via biomimetic self-assembly. These hierarchical structures combined goat anti-mouse IgG-conjugated horseradish peroxidase (HRP) and bovine serum albumin (BSA) with copper phosphate, forming BSA-(IgG-HRP)-Cu_3_(PO_4_)_2_ nanocomposites. Implemented in nano-ELISA platforms, this system achieved an LOD of 3.90 ng/mL for paraben detection.

Magnetic nanomaterials offer additional advantages, such as facilitating enzyme immobilization and enabling regeneration. Gan et al. [24] developed a reusable enzymatic biosensor using Fe_3_O_4_/Au magnetic nanoparticles (GMPs) functionalized with AChE on screen-printed carbon electrodes (SPCEs). This sensor integrates Nano-ZrO_2_, Prussian Blue (PB), and Nafion membranes with a magnetic Au core–shell design. Fe_3_O_4_ enables rapid enzyme separation (<30 s) and Au improves AChE biocompatibility (85% activity retention). The superparamagnetic GMPs also allow for a 95% regeneration of the sensor surface. This sensor demonstrated a linear detection range of 1.0 × 10^−3^–10 ng/mL, an LOD of 5.6 × 10^−4^ ng/mL, and a response time under 15 s with high specificity. Table 2 summarizes representative enzyme-based nanosensors for pesticide detection.

### 2.2. Antibody

Antibodies are among the most widely used biorecognition elements in biosensor development, and sensors utilizing them are known as immunosensors. Their high affinity and specificity toward target analytes make them highly effective in complex sample matrices. Immunoglobulin G (IgG) is the most commonly used class of antibody. Based on selectivity and production method, antibodies are categorized as polyclonal (pAb), monoclonal (mAb), or recombinant antibodies (rAb).

Despite their advantages, antibodies face challenges such as poor solubility, low thermal stability, aggregation at elevated temperatures, and loss of binding affinity under harsh conditions. To address these limitations, smaller recombinant antibody fragments, including Fab, Fv (variable fragment), scFv (single-chain variable fragment), and single-domain antibody (sdAb, or nanobody), have been developed for biosensor fabrication [41].

Most pesticides are haptens, which are small molecules that elicit an immune response only when conjugated to a larger carrier molecule such as bovine serum albumin (BSA). Numerous antibodies are now commercially available for specific pesticides across various classes, including organophosphates, organochlorines, pyrethroids, carbamates, and triazines. A novel approach in antibody development is the use of broad-specificity antibodies, which can detect multiple structurally similar analytes within a single assay [42]. These are generated using multi-hapten antigens or generic haptens to target compound classes.

Combining antibodies with magnetic nanomaterials and nanoenzymes significantly enhances sensor performance. Chen et al. [43] developed a biosensor for organophosphorus pesticide detection using bimetallic nanoenzymes and immunomagnetic separation. This platform employs complementary DNA-conjugated gold nanoparticles (AuNPs) bound to catalytic Au@Pt nanoenzymes that compete with magnetic nanoparticle probes (functionalized with OVA-hapten conjugates) for antibody binding. After magnetic separation, residual Au@Pt nanoenzymes catalyze Amplex™ Red oxidation to fluorescent resorufin in thiol-functionalized microplates. This dual-amplification system achieved LODs of 9.88, 3.91, and 1.47 ng/kg for parathion, triazophos, and chlorpyrifos, respectively.

**Figure 5:**
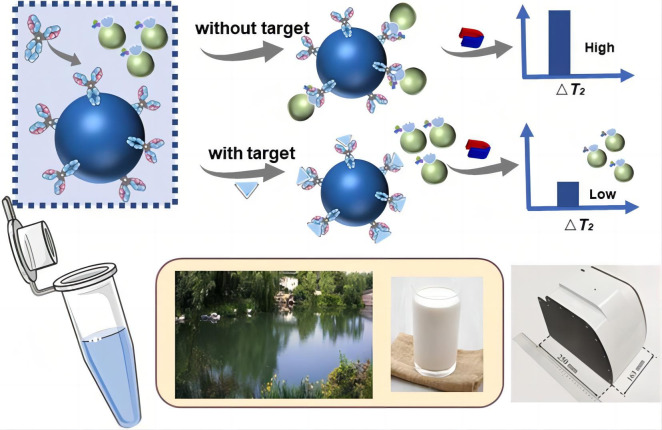
The work principle of the immunogold-functionalized magnetic nanoparticles as magnetic relaxation switching probes [32]. Adapted with permission from ref. [32]. Copyright 2023, Elsevier.

Hou et al. [32] developed a multiplex magnetic relaxation switching (MRS) biosensor using CL-CN/1D2 broad-spectrum antibodies and immunogold-functionalized graphene magnetic nanoparticles (GMNs) as MRS probes. This sensor enabled the simultaneous detection of eight pyrethroid pesticides in lake water and milk with LODs ranging from 2.72 to 4.42 µg/L (Figure 5).

**Figure 6:**
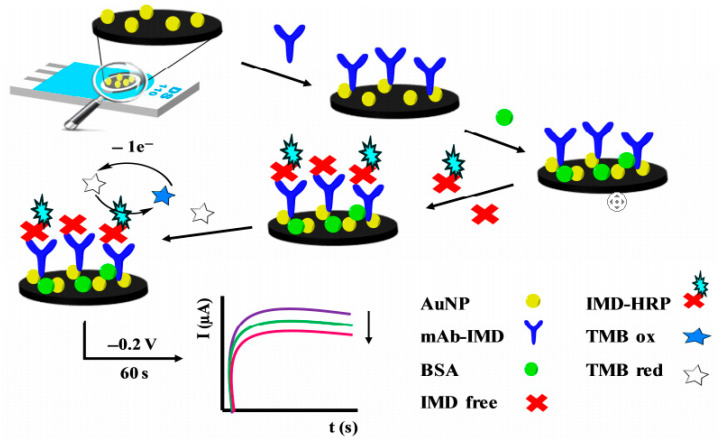
Scheme of the direct competitive immunosensor for the detection of imidacloprid (IMD) on AuNP-SPCE using monoclonal antibodies [44]. The colored line represents the response current of the screen-printed electrode. Adapted with permission from ref. [44]. Copyright 2020, Elsevier.

Pérez Fernández et al. [44] constructed a competitive immunosensor using AuNP-functionalized screen-printed carbon electrodes (AuNP-SPCEs) for imidacloprid detection. Monoclonal antibodies immobilized on AuNP-SPCEs bind free imidacloprid (IMD) in competition with HRP-conjugated IMD (IMD-HRP). The resulting HRP-catalyzed oxidation of TMB, followed by electrochemical reduction at the SPCE, produces a catalytic current inversely proportional to IMD concentration. This system showed a linear range of 50–10,000 pmol/L and an LOD of 2.1 pmol/L (Figure 6).

MXene-based materials offer excellent biocompatibility for antibody immobilization. An MXene-rGO/ED-Ab/FTO immunosensor was developed for endosulfan detection, combining MXene’s bioaffinity with reduced graphene oxide (rGO)’s protective properties [45]. The hybrid material was deposited on fluorine-doped tin oxide (FTO) electrodes, resulting in a stable functional interface compatible with aqueous matrices. The sensor exhibited a broad linear detection range (0.1 ppt–1 ppm) and an LOD of 0.497 ppt, and was validated in environmental water and plant tissues.

**Figure 7:**
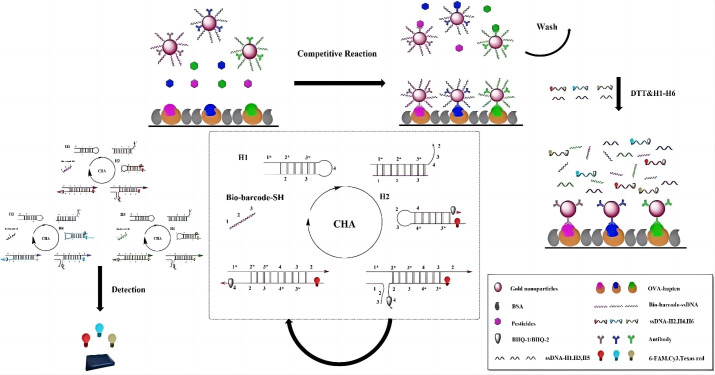
Schematic of the competitive fluorescent immunosensors for OP detection [46]. Adapted with permission from ref. [46]. Copyright 2023, Elsevier.

Wang et al. [46] designed a dual-signal amplification strategy by integrating catalytic hairpin assembly (CHA) with competitive immunofluorescence. This method used fluorescent hairpin DNA probes for cascaded signal enhancement, enabling the simultaneous detection of triazophos, parathion, and chlorpyrifos with detection ranges of 0.01–50 ng/mL and LODs of 0.012, 0.0057, and 0.0074 ng/mL, respectively (Figure 7).

### 2.3. Aptamer

Aptamers are synthetic single-stranded DNA, RNA, or peptides generated via the SELEX (Systematic Evolution of Ligands by Exponential Enrichment) process, which allows them to bind specific targets with high affinity and structural adaptability, akin to antibodies. Their advantages include structural flexibility, rapid in vitro generation, high stability, low immunogenicity, and compatibility with label-free detection.

Aptamer-based sensors utilize signal transduction mechanisms such as conformational changes, redox behavior, or optical modulation, and have been applied in chemiluminescent-, colorimetric-, and fluorescence-based pesticide detection platforms.

Jiang et al. [47] developed a multiplex fluorescence assay using a 6-FAM-labeled aptamer with conserved organophosphorus-binding sequences, coupled with functionalized magnetic nanoparticles for separation. This system enabled the simultaneous detection of trichlorfon, glyphosate, and malathion, with LODs of 72.20 ng/L, 88.80 ng/L, and 195.37 ng/L, respectively, across a linear range of 0.0001–10 mg/L. The assay was successfully applied to lettuce and carrot samples.

Bala et al. [48] designed a simple label-free colorimetric biosensor for methyl phosphate detection using aptamer-coated AuNPs. In the absence of the target pesticide, the aptamer maintains a flexible structure that stabilizes AuNPs, keeping the solution red. Upon target binding, the aptamer folds, causing AuNP aggregation and a visible color change from red to blue. This biosensor demonstrated a detection range of 0.01 nM to 1.3 µM with an LOD of 0.01 nM.

### 2.4. Molecularly Imprinted Polymer (MIP)

MIPs are synthetic polymers with specific recognition sites complementary in shape, size, and functional groups to a target molecule, functioning as “artificial antibodies”. They offer high selectivity, rapid and low-cost synthesis, and excellent chemical and thermal stability, but typically exhibit lower binding affinity compared to natural antibodies [41,49].

Combining MIPs with surface plasmon resonance (SPR) technology can significantly enhance selectivity. Do et al. [50] developed a glyphosate-specific SPR biosensor using spin-coated chitosan/ZnO or chitosan/graphene oxide films, achieving an LOD of 8 nM. Saylan et al. [51] constructed a multiplex MIP-SPR sensor for the simultaneous detection of cyanine, simazine, and atrazine, with LODs of 95, 31, and 91 ppm, respectively.

Gokturk et al. [52] synthesized hydroxyethyl methacrylate N-methylpropenyl-(L)-phenylalanine methyl ester nanoparticles (MIP@NPs) as glyphosate-specific imprints, forming a highly selective plasma nanosensor for glyphosate. The sensor showed 4.95- and 3.92-fold higher selectivity for glyphosate over malathion and malaoxon, respectively, and achieved an LOD of 0.120 ppb over a linear range of 0.001–10 ppm. It was successfully applied to human urine samples.

Oymen et al. [53] developed a coumaphos-imprinted SPR nanosensor for honey sample analysis, achieving a wide linear range of 0.1–250 ppb and an ultralow LOD of 0.001 ppb.

## 3. Transducers of Nanosensors for Pesticide Detection

Besides the receptor, the transducer or sensing element is another major component of a sensor. Based on their transduction mechanisms, biosensors can be categorized as electrochemical, optical, thermometric, or piezoelectric types [54,55,56]. Among these, electrochemical and optical sensors have been the most extensively studied for pesticide detection. Therefore, this section summarizes their applications in pesticide detection in agricultural products, focusing primarily on electrochemical and optical biosensors.

### 3.1. Electrochemical Biosensors

Electrochemical biosensors are analytical devices that convert chemical interactions between target analytes and recognizers into measurable electrical signals. These sensors use electrodes as transducers, with surface-modified functional materials acting as biorecognition elements. By measuring electrical parameters (e.g., voltage, current, resistance), they establish quantifiable correlations between analyte concentrations and electrical responses, enabling sensitive and specific detection [57]. Nanomaterial-functionalized electrodes (e.g., graphene oxide, AuNPs, AgNPs) offer increased electroactive surface areas, thereby significantly enhancing sensitivity and attracting increasing research interest. Based on the measured parameter, electrochemical sensors can be further divided into potentiometric, amperometric, and impedance biosensors.

#### 3.1.1. Potentiometric Biosensor

Potentiometric biosensors, a subtype of electrochemical sensors, quantify analytes by measuring potential differences between a working and a reference electrode under zero-current conditions. Various types of potentiometric biosensors have been used to detect pesticides in agricultural products.

By depositing enzymes or redox-sensitive materials onto the working electrode, the detection of target substances can be achieved. Most AChE-based sensors function via enzyme inhibition: AChE catalyzes the hydrolysis of acetylthiocholine chloride (ATCl) to thiocholine (TCh), which produces a redox-active product. Organophosphate pesticides (OPs) inhibit AChE, altering the oxidation current. For example, a potentiometric biosensor employing an AChE-functionalized Ag/rGO/chitosan nanocomposite film was developed for carbaryl detection. This sensor showed a wide linear range (1.0 × 10^−8^ to 1.0 μg/mL) and a low detection limit (LOD) of 1.0 × 10^−9^ μg/mL [35].

Liu et al. [33] developed a novel AChE biosensor using a GCE modified with AgNPs, carboxymethyl graphene (CGR), and Nafion, where CS was used to immobilize AChE, enhancing its activity and stability. This biosensor exhibited excellent performance with LODs of 5.3 × 10^−14^ M for chlorpyrifos and 5.45 × 10^−13^ M for carbaryl.

Other enzymes besides AChE have also been employed. For instance, Chetana Vaghella et al. developed a urease-based biosensor using AuNPs embedded in agarose-guar gum, achieving an LOD of 0.5 ppm for glyphosate and excellent stability over 180 days [36].

Although most electrochemical sensors operate based on enzyme inhibition, other mechanisms also exist. Tümay et al. [58] developed a dual-analyte sensor using ferrocene-thiophene-modified CNTs (FT@CNT) to simultaneously detect parathion and chlorantraniliprole in tomatoes and apples, achieving LODs of 5.3 nmol/L and 8.1 nmol/L, respectively. Similarly, Rashed et al. [59] constructed a ternary nanocomposite Ag@Meso-C/Fe_2_O_3_ sensor for selective imidacloprid detection with an LOD of 1.06 μM, while Chen et al. [60] used Au@MWCNTs/GCE nanocomposites for detecting dichlorvos with a 5 nM LOD in leafy vegetables.

#### 3.1.2. Amperometric Biosensors

Amperometric biosensors measure current changes at a constant applied potential, correlating the current-to-analyte concentration [61,62,63]. The rise of screen-printed electrodes (SPEs)—owing to their low cost, portability, and ease of mass production—has significantly boosted research interest in this area.

SPEs are fabricated via the template-based deposition of conductive inks on flexible substrates (e.g., PET, paper). They offer reproducibility, low cost, and multiplexing capability [64,65]. Nanomaterials further enhance their performance through increased conductivity, surface area, and catalytic activity.

**Figure 8:**
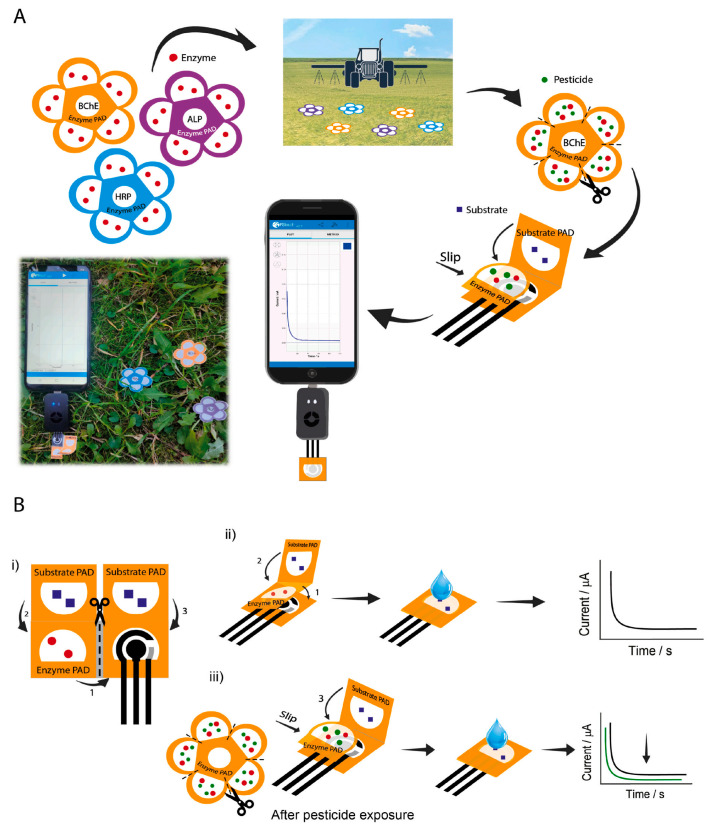
(**A**) The flower-like origami-paper-based electrochemical device able to detect pesticides using a smartphone-assisted potentiostat. (**B**) Schematic representation of the origami-paper-based electrochemical biosensor composed by an office-paper-based screen-printed electrode and three filter PADs (i), mechanism of measurement of the initial enzymatic activity in absence of pesticide (ii), and of the residual enzymatic activity after exposure to pesticide solution (iii) [66]. Adapted with permission from ref. [66]. Copyright 2022, Elsevier.

Caratelli et al. [66] designed a flower-shaped paper SPE and filter paper pads loaded with enzymes and enzyme substrates for detecting three pesticide residues in the aerosol phase (Figure 8). The paper-based electrochemical platform detects paraoxon, 2,4-dichlorophenoxyacetic acid (2,4-D), and glyphosate at ppb levels by quantifying their inhibition of three enzymes: butyrylcholinesterase, alkaline phosphatase, and peroxidase. This integrated device consists of three office-paper-based SPEs and filter paper pads preloaded with enzymes and substrates. Detection involves the chronoamperometric measurement of initial and residual enzymatic activity using a smartphone-assisted potentiostat, with inhibition percentages (proportional to aerosolized pesticide concentrations) as the analytical signal. The device achieved detection limits of 30 ppb (2,4-D), 10 ppb (glyphosate), and 2 ppb (paraoxon) in the aerosol phase. This sensor combines paper-based microfluidics, enzyme inhibition mechanisms, and portable electrochemical detection into a unified system, delivering an efficient and practical solution for the on-site monitoring of pesticide-contaminated aerosols.

To enhance sensitivity, Tang et al. [67] combined SERS with SPEs, creating a dual-channel sensor for acetamiprid with an LOD of 0.04 μM, over sevenfold lower than traditional SERS methods.

Sharifi et al. [37] developed a photoelectrochemical sensor using nanocellulose paper immobilized with BChE, allowing for OP detection with real-time smartphone readouts. This system achieved detection within the 20–100 ppb range and validated its use in wastewater.

Non-enzymatic sensors are also emerging. Unnikrishnan et al. [68] constructed an MnO_2_-GNP-based SPCE sensor for carbaryl detection with an LOD of 0.07 μM, outperforming enzymatic systems. Another SPE using an Fe–Ni alloy achieved phosmet detection in olive oil with an LOD of 0.1 nM [69].

Dhull et al. [30] employed a CNT-AuNP composite for an OP biosensor with a rapid response (10 s) and multi-cycle reuse, achieving LODs as low as 1.9 nM across several OPs.

Electrostatic charge changes from enzyme reactions also offer sensing potential. A AuNPs/MPS/Au biosensor used charge inversion for carbaryl detection with a 1 nM LOD and high cost-efficiency [38].

Three-dimensional graphene architectures, such as 3DG-CuO nanoflowers, offer enhanced immobilization surfaces. Bao et al. [39] reported a sensor achieving an LOD of 0.92 pM for malathion in environmental samples.

**Figure 9:**
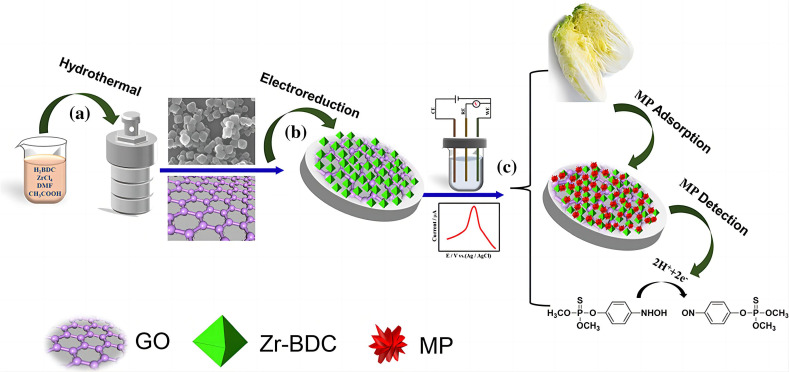
Schematic description of the synthesis of Zr-BDC via a hydrothermal method (**a**), preparation of Zr-BDC-rGO nanocomposites on GCE byelectro-reduction (**b**), and the construction of the proposed pesticide electrochemical sensor and detection of MP mechanism (**c**) [70]. Adapted with permission from ref. [70]. Copyright 2021, Springer.

Similarly, a Zr-based MOF-rGO composite achieved an LOD of 0.5 ng/mL for methyl parathion with excellent anti-interference performance in cabbage samples [70] (Figure 9).

#### 3.1.3. Impedance Biosensor

Impedance biosensors detect electrochemical impedance changes resulting from analyte–receptor interactions. These changes alter the electrical double layer at the electrode–solution interface. Their key advantages include label-free detection, high sensitivity, rapid response, and ease of miniaturization. However, limitations include sensitivity to environmental noise, limited reproducibility, and complex sample matrix compatibility.

Zhao et al. [71] used microelectrode arrays functionalized with chlorpyrifos antibodies and achieved an LOD of 1 ng/mL. For chiral pesticide detection, Zhang et al. [40] developed a biosensor using reduced GO for the enantiomer discrimination of methamidophos, with LODs three orders of magnitude lower than CD spectroscopy. Portable smartphone integration allowed for on-site detection at 1 V, offering practical field application potential.

In summary, electrochemical biosensors offer (1) high sensitivity, (2) miniaturization compatibility, (3) low cost, (4) rapid response, (5) user-friendly operation, and (6) immunity to optical interference. However, stability issues of bioreceptors, matrix interferences, and cross-reactivity remain key challenges to be addressed. Table 3 summarizes representative electrochemical nanosensors and their applications in pesticide detection.

### 3.2. Optical Biosensor

An optical biosensor is an analytical device that integrates biorecognition elements with optical transduction technology. It enables the real-time monitoring of biomolecular interactions with high sensitivity and specificity by detecting changes in light intensity, wavelength, or other optical properties during the binding of probes to target molecules [75]. In pesticide residue detection, fluorescent biosensors are predominant due to their reliance on measurable fluorescence signal shifts triggered by target–probe binding. Other optical biosensing techniques include surface plasmon resonance (SPR), photoluminescence, refractive index modulation, and Raman scattering spectroscopy (e.g., SERS) [76].

#### 3.2.1. Fluorescent Biosensor

Fluorescent biosensors detect analytes through target-induced fluorescence changes in fluorophore emission intensity (quenching, enhancement, or wavelength shift) that are proportional to the analyte concentration. Compared to traditional methods, their main advantages include ultrahigh sensitivity and selectivity, along with the capability for on-site and real-time detection. Fluorescent labels are attached to recognition elements (commonly enzymes, antibodies, or aptamers). When target molecules bind to these recognition elements, changes occur in the microenvironment of the fluorescent molecules, generating detectable fluorescent signals. Fluorescent biosensors commonly utilize labeling materials such as quantum dots (QDs) and fluorescent dyes. Of these, QDs have emerged as the predominant choice due to their superior photostability, tunable optical properties, biocompatibility, and extended fluorescence lifetimes. The sensing mechanism includes fluorescence resonance energy transfer (FRET), upconversion luminescence, and other related phenomena.

**Figure 10:**
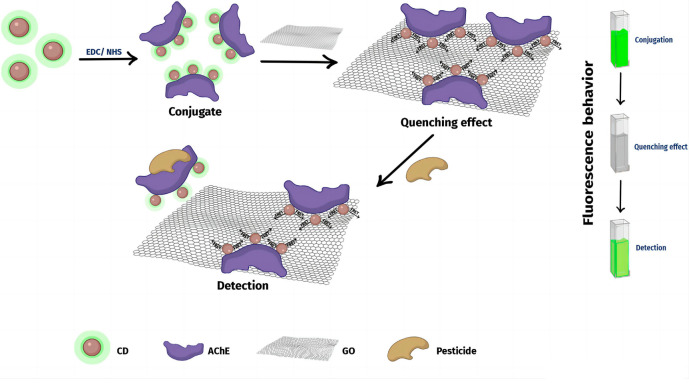
The detection mechanism of the proposed biosensor showing the quenching of the fluorescence for the CDs conjugated with AChE in the presence of GO, and the recovery of the fluorescence signal in the presence of the pesticide [25]. Adapted with permission from ref. [25]. Copyright year 2022, Frontiers.

Gaviria et al. [25] established a fluorescent biosensor combining AChE as a biological probe, carbon dots (CDs) as fluorophores, and graphene oxide (GO) as a fluorescence quencher (Figure 10). Covalent bonding between AChE and CDs reduces fluorescence intensity by approximately 57% through GO-induced quenching, primarily governed by FRET. Pesticide exposure induces irreversible AChE inhibition and conformational changes, enabling π-π interactions between the AChE–pesticide complex and GO, which detaches CDs from GO, restoring fluorescence emission. The biosensor detects chlorpyrifos in the linear range of 1–25 nM, with a limit of detection (LOD) of 0.14 ppb in pure form and 2.05 ppb in commercial Lorsban^®^, demonstrating successful application in spiked tap water analysis and long-term storage stability (25 days).

Chandra et al. [26] developed fluorescent Jatropha-derived carbon quantum dots (J-CQDs) via one-step hydrothermal synthesis from Jatropha curcas fruit. These J-CQDs exhibit bright-blue fluorescence (emission at 440 nm). The detection mechanism exploits the AChE-mediated conversion of ACh to TCh, which reacts with Ellman’s reagent (DTNB) to generate yellow 5-thio-2-nitrobenzoic acid (TNBA). TNBA effectively quenches J-CQD fluorescence through electron transfer. Chlorpyrifos inhibits AChE activity, preventing TNBA formation and restoring fluorescence intensity. The sensor achieves a chlorpyrifos detection limit of 2.7 ng/mL, demonstrating its practical utility in agricultural sample analysis.

**Figure 11:**
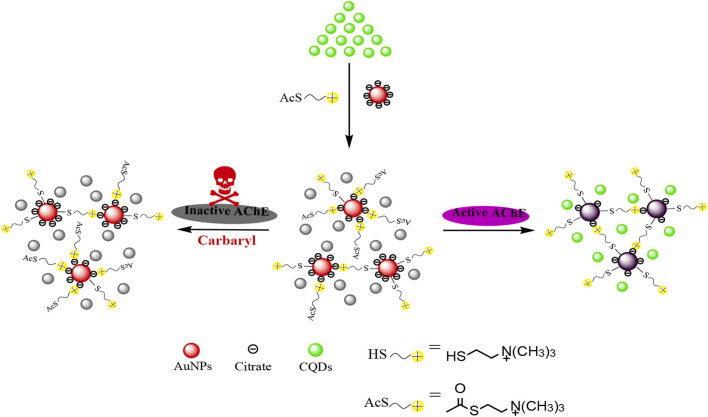
Schematic diagram of dual-signal analysis of carbaryl [27]. Adapted with permission from ref. [27]. Copyright 2020, Elsevier.

Chen et al. [27] developed a dual-signal biosensor using B, N-doped CQDs, and AuNPs for carbaryl detection (Figure 11). This FRET-based system eliminates the inner filter effect and bypasses the need for direct fluorophore–quencher interactions. The mechanism relies on the AChE-catalyzed hydrolysis of acetylthiocholine iodide (ATC) to TCh, which induces AuNP aggregation and subsequent CQD fluorescence recovery at 490 nm. Carbaryl inhibits AChE activity, preventing TC generation and maintaining dispersed AuNPs, thereby reducing fluorescence restoration. The dual readout system combines CQD fluorescence (0.2–20 μg/L detection range) with AuNPs absorbance (linear concentration dependence), achieving reliable carbaryl quantification in environmental water samples.

**Figure 12:**
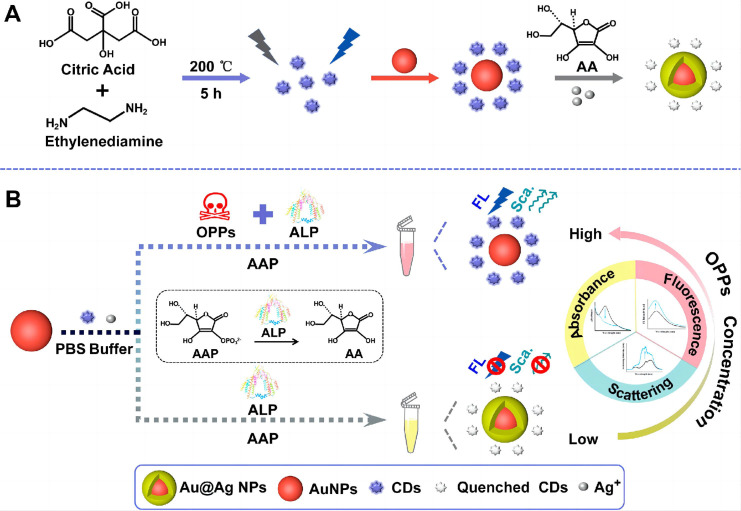
Schematic illustrations of the synthesis of CDs and formation of a Au@Ag nanostructure (**A**) and the triple-signal sensing platform for the assay of OPs (**B**) [77]. Adapted with permission from ref. [77]. Copyright 2022, American Chemical Society.

Monitoring changes in multiple optical detection signals triggered by target analytes improves detection sensitivity, reliability, and specificity. Liu et al. [77] engineered a multi-signal detection platform by integrating silver-deposited AuNPs with CDs in phosphate-buffered saline (AuNPs/Ag^+^/CDs/PBS) (Figure 12). In this system, alkaline phosphatase (ALP) converts endogenous substrates (2-phospho-L-ascorbicacid trisodium salt) to L-ascorbic acid (reducible), triggering silver ion reduction on AuNP surfaces and forming a Au@Ag core–shell nanostructure, resulting in the quenching of fluorescence, shift of resonance absorption, and decrease in Rayleigh scattering. OPs were quantified through ALP activity inhibition, which modulates the composite’s UV-vis absorption, fluorescence intensity, and Rayleigh scattering signals. The nanosensor achieved an LOD of 10 ng in the line range of 0 to 2.1 μg/mL for acephate. This tri-modal approach enabled the simultaneous colorimetric, fluorometric, and resonance Rayleigh scattering detection of OPs, and was successfully validated in vegetable samples.

QDs also enable sensitive biosensors through immunoreaction integration. Liao et al. [78] developed a QD-based ELISA using CdSe/ZnS QDs conjugated with monoclonal antibodies (mAbs) for triazophos detection. The method employs competitive binding: triazophos competes with ovalbumin for mAb binding sites on QDs. After washing, the retained antigen–antibody–QD complexes exhibit measurable fluorescence intensity, achieving an LOD of 0.508 ng/L for triazophos.

Besides FRET, upconversion luminescence is also used in fluorescent sensing. Wang et al. [79] developed a non-enzymatic ratiometric nanosensor for OP detection using upconversion luminescence. The system employs Tween-20-modified blue-emissive upconversion nanoparticles (Tween 20-UCNPs-HODN), where the 475 nm emission is quenched via luminescence resonance energy transfer (LRET) to pesticide probes (HODN). OP exposure diminishes the probe’s UV absorption, suspending LRET and rapidly restoring UCNP luminescence. This turn-on mechanism enables dimethoate detection across a linear range of 0–80 μM with an LOD of 0.14 μM.

Luo et al. [80] prepared a turn-on fluorescence sensor using rhodamine B-functionalized Ag/Au bimetallic nanoparticles (RhB-Ag/AuNPs) that leverages competitive metal-ligand coordination dynamics. In the native state, RhB’s fluorescence remains quenched through plasmonic energy transfer to the nanoparticles. Upon exposure to OPs, the stronger thiophosphate–Ag/Au binding affinity triggers RhB displacement, restoring fluorescence intensity proportionally to OP concentration. This coordination-driven mechanism enabled ultrasensitive OP detection across 3.3–280 pg/mL with an LOD of 1.8 pg/mL. The sensor was successfully applied to OPs detected in apple and groundwater samples.

Zhang et al. [81] engineered peptide nanodot-bridged MOFs (PNMOFs) through the Zn^2+^-mediated coordination of tryptophan-phenylalanine dipeptide nanodots and dihydroxyhydroxamic acid linkers for ultrasensitive pyrethroid detection. The framework’s fluorescence undergoes cyhalothrin-induced quenching via PET, achieving 0.34 μg/L detection limits within 20 s. This cascade amplification platform enables the smartphone-coupled fluorescence imaging of both dissolved and surface-adsorbed pyrethroids through a paper-based microfluidic interface, demonstrating 97% accuracy in tea leaf analysis compared to GC-MS. The modular design permits over 50 regeneration cycles with <5% signal loss, outperforming conventional antibody-based assays in cost (83% reduction) and field deployability.

Due to their significant advantages, carbon quantum dots (CQDs) have been widely used as photosensitive materials. Sinha et al. [82] synthesized CQDs from waste tea leaves through a one-step hydrothermal method, achieving a 40.05% quantum yield. These CQDs enabled the FRET-based detection of three pesticides: quinalphos 25 EC (turn-on response), thiamethoxam 25 WG (turn-off), and propargite 57EC (turn-off). The system demonstrated LODs of 0.2, 1, and 10 ng/mL, respectively, within a linear range of 0.2–5000 ng/mL, using a fluorescence switching mechanism.

Fluorescent nanocomposites with multicolor emissions enable reliable and sensitive pesticide detection. Chen et al. [83] developed a dual-emission sensor using blue-emitting nitrogen-doped CQDs (N-CQDs) and red-emitting copper nanoclusters (CuNCs). Through solvent modulation, this system selectively detects dithiocarbamoyl and paraquat pesticides with LODs of 7.49 nM and 3.03 nM, respectively. A smartphone-based colorimetric platform was integrated for the rapid on-site visual monitoring of pesticide residues in food samples.

A single QD system enables multiplex pesticide detection through distinct fluorescence responses. Chen et al. [84] fabricated cadmium sulfide quantum dots (CdS QDs) via one-step synthesis for the simultaneous detection of dichlorvos, paraquat, and glufosinate ammonium. The QDs exhibited three discriminative signals: paraquat-induced fluorescence quenching, dichlorvos-triggered 30-fold fluorescence enhancement, and glufosinate ammonium-induced 150 nm blue shift with fluorescence amplification. Detection limits reached 0.23 mM (dichlorvos), 1.44 μM (paraquat), and 49.8 μM (glufosinate ammonium). The system was successfully integrated with smartphone-based detection for on-site water monitoring.

Zhang et al. [85] created a fluorescent OPCD@UiO-66-NH_2_ composite by encapsulating ortho-phenylenediamine-derived carbon dots (OPCD) within UiO-66-NH_2_ MOF channels. This hybrid material combines the strong luminescence of OPCD with the porous structure of UiO-66, demonstrating stable aqueous fluorescence. The sensing mechanism involves Cu^2+^-induced fluorescence quenching through non-fluorescent ground-state complex formation (OPCD@UiO-66-NH_2_–Cu^2+^). Quinalphos disrupts this complexation, restoring fluorescence intensity via competitive binding with Cu^2+^. This dual-responsive system enables sequential detection of Cu^2+^ (0–50 μM) and quinalphos (0–16 μM) with an LOD of 0.3 nM for quinalphos. The ratiometric fluorescence response showed 93% recovery in spiked agricultural samples, confirming practical detection capability.

Bera et al. [86] made a fluorescent OPCD@UiO-66-NH_2_ composite by encapsulating ortho-phenylenediamine-derived carbon dots (OPCD) within UiO-66-NH_2_MOF channels. This hybrid material combines the superior luminescence of OPCD with the porous architecture of UiO-66, demonstrating stable aqueous fluorescence. The sensing mechanism involves Cu^2+^-induced fluorescence quenching through non-fluorescent ground-state complex formation (OPCD@UiO-66-NH_2_-Cu^2+^). Quinalphos disrupts this complexation, restoring fluorescence intensity via competitive binding with Cu^2+^. This dual-responsive system enables the sequential detection of Cu^2+^ (0–50 μM) and quinalphos (0–16 μM) with a quinalphos detection limit of 0.3 nM. The ratiometric fluorescence response shows 93% recovery in spiked agricultural samples, confirming practical detection capability.

Nethaji et al. [87] engineered blue-emitting carbon dots (B-CDs, QY = 49.3%) for the multiplex detection of four organochlorine pesticides: heptachlor (HEP), endosulfan (ENS), chlormephos (CDF), and 2,4-DPAC. The amino-functionalized surface enables differential fluorescence responses through Cl/OH group interactions: HEP enhances emission via 23% FRET, while 2,4-DPAC quenches fluorescence by 68% through electron transfer. ENS and CDF exhibit intermediate signal modulation (41% and 54%, respectively). The sensor achieves LODs of 2 nM (HEP), 99 nM (ENS), 160 nM (CDF), and 82 nM (2,4-DPAC), validated in spiked agricultural runoff samples. This ratiometric approach permits simultaneous quantification by correlating emission intensity patterns with specific pesticide concentrations.

**Figure 13:**
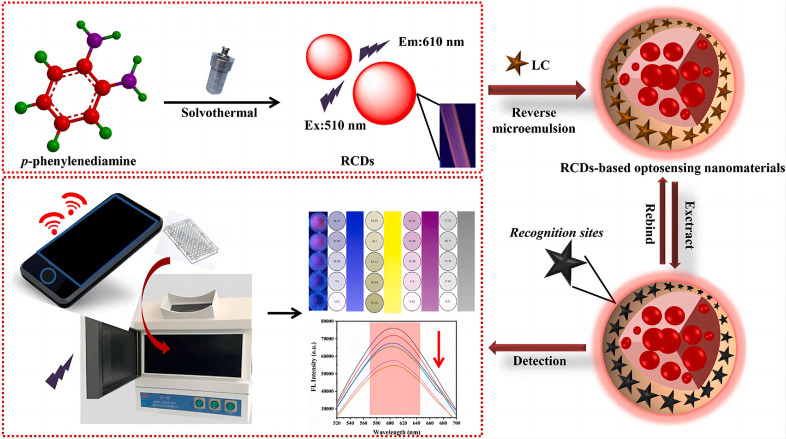
Schematic illustration of the platform comprising RCD-based optosensing nanomaterials for the detection of Lambda-cyhalothrin(LC) [88]. Adapted with permission from ref. [88]. Copyright 2021, Elsevier.

Zhu et al. [88] constructed a smartphone-integrated optical sensor using amino-functionalized red-emissive carbon dots (RCDs, λ_em = 625 nm) for on-site pyrethroid monitoring (Figure 13). The system exploits λ-cyhalothrin’s selective fluorescence quenching via PET between pesticide amino groups and RCD surface states, achieving an LOD of 0.89 μg/L within a 2 min response. This ratiometric platform enables the real-time field analysis of 1–120 μg/L pyrethroids (lambda-cyhalothrin, LC) in tea extracts via a colorimetric app, demonstrating 96% correlation with HPLC in 38 commercial samples. The RCDs maintain 95% signal stability after 15 regeneration cycles, outperforming commercial test strips in cost (78% reduction) and inter-analyte selectivity (<8% cross-reactivity).

#### 3.2.2. Surface Plasmon Resonance (SPR) Biosensor

An SPR arises from coherent electron oscillations at metal–dielectric interfaces. These phenomena enable sub-diffraction limit field confinement and local electromagnetic field enhancement. The detection mechanism relies on three critical phenomena: surface plasmon wave generation, resonance coupling, and refractive index sensitivity. SPR has been widely used for environmental pollution monitoring, food safety, and biomedical diagnostics [53]. Emerging designs using hyperbolic metamaterials (h-BN/graphene heterostructures) and Fano-resonant nanostructures further push sensitivity to single-molecule levels [89].

**Figure 14:**
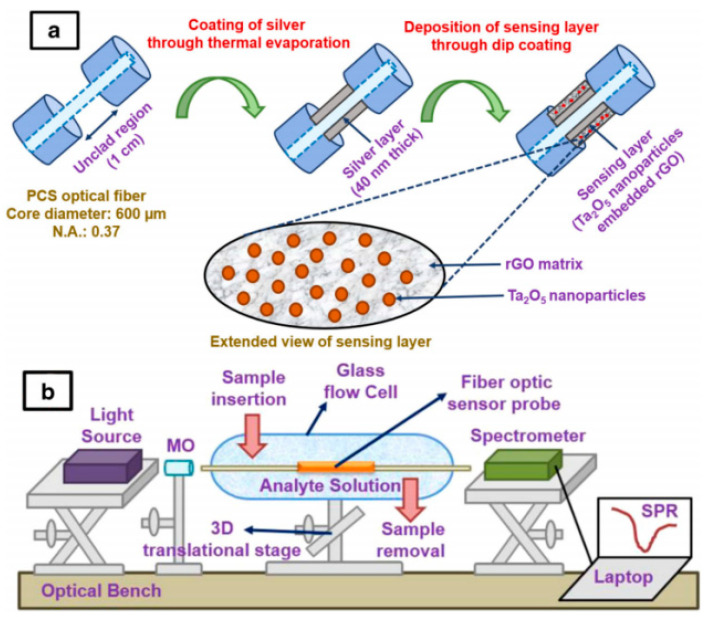
(**a**,**b**) Schema of SPR-based fiber-optic nanosensor for the pesticide fenitrothion utilizing Ta_2_O_5_ nanostructures sequestered onto a reduced GO matrix [90]. Adapted with permission from ref. [90]. Copyright 2020, Springer.

Using optical fiber as the SPR substrate can significantly improve real-time monitoring capability. Kant et al. [90] developed an SPR-based fiber-optic nanosensor for fenitrothion detection (Figure 14). The sensor operates via refractive index modulation at a sensing interface comprising Ta_2_O_5_ nanoparticles embedded in a reduced graphene oxide (rGO) matrix deposited on a silver-coated optical fiber core. Analyte interaction alters the interface’s refractive index, enabling the detection of imidacloprid in a linear range of 0.25–4 μM with an LOD of 38 nM and 23 s response time. The sensor exhibits high selectivity and specificity and excellent repeatability. The fiber-optic design permits real-time analysis and remote monitoring, demonstrating applicability for on-site pesticide detection.

The advantages of SPR biosensors include (1) label-free operation, (2) real-time and sensitive detection, (3) a broad dynamic range, and (4) versatile compatibility. The limitations of SPR biosensors are (1) high costs, (2) technical expertise is required, and (3) susceptibility to environmental factors. Current technological bottlenecks and future challenges for SPR include (1) cost control, (2) standardized production processes, (3) complex sample adaptation, (4) miniaturization and multi-channel detection, (5) integration of AI data analysis technologies, and (6) live monitoring capabilities. Future directions for SPR biosensor development are (1) enhancements in sensitivity and resolution, (2) high-throughput capabilities, (3) miniaturization and portability, (4) advanced material synergies, (5) AI-driven analytics, and (6) multimodal integration.

#### 3.2.3. Surface-Enhanced Raman Scattering (SERS) Biosensor

In recent years, vibrational spectroscopy techniques, particularly Raman spectroscopy, have gained significant attention in biomedical research, environmental monitoring, pesticide residue detection, and renewable energy applications due to their rapid and label-free analytical capabilities [91,92,93]. The advancement of plasmonics and nanotechnology has driven the emergence of SERS. This technique immobilizes samples on nanostructured metal substrates (e.g., colloidal gold/silver nanoparticles or solid metallic nanostructures) to monitor Raman signal modulations induced by target–substrate interactions. Through the rational selection of surface enhancers and substrates, SERS amplifies Raman scattering signals by 10^10^–10^15^ times compared to conventional Raman spectroscopy, enabling the non-destructive rapid analysis of trace and ultratrace samples in complex matrices [94,95].

SERS enhancement primarily arises from two synergistic mechanisms: electromagnetic enhancement (excitation of localized surface plasmon resonance, dominant contribution) and chemical enhancement (molecular orbital hybridization, secondary contribution). This technique’s capacity for multiplexed analysis in complex biological systems underscores its transformative potential in diagnostics and analytical sciences. Portable SERS systems incorporating flexible substrates and tailored nanofabrication techniques have emerged as preferred tools for on-site pesticide detection.

**Figure 15:**
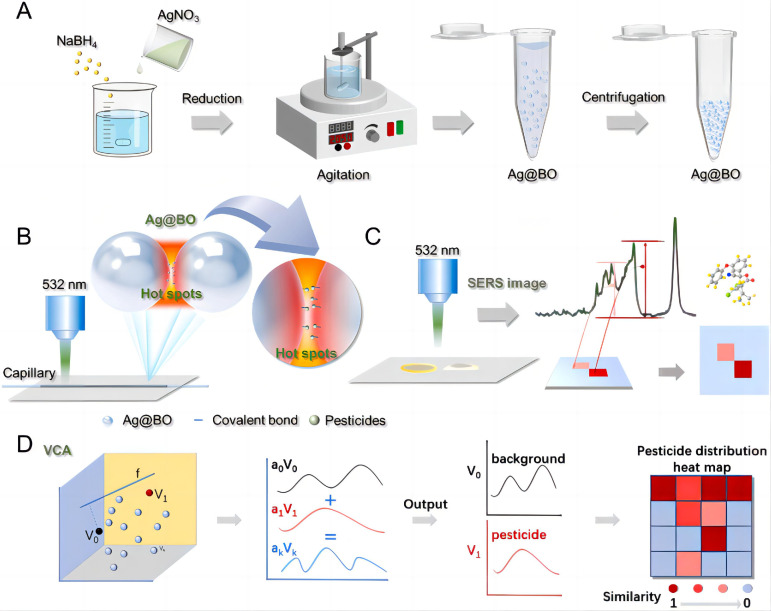
Schematic representation of the whole process of monitoring pesticide application. (**A**) Detailed preparation flow chart of Ag@BO nanoparticle substrate. (**B**) Schematic diagram of SERS detection principle and “hot spots” based on Ag@BO nanoparticle substrate. (**C**) SERS imaging of pesticides on pericarp and in flesh obtained using the current method. (**D**) Principle of SERS imaging analysis method: vertex component analysis (VCA) [97]. Adapted with permission from ref. [97]. Copyright 2024, Elsevier.

Silver nanomaterials have the strongest plasmonic response but are less stable than gold nanomaterials, while gold–silver bimetallic materials exhibit synergistic effects and stronger enhancement. Wang et al. [96] prepared a SERS method based on silver nanoparticles (AgNPs) for detecting pesticide residues in apple samples, achieving LODs of 1.28 × 10^−9^ mol/L and 2.47 × 10^−10^ mol/L for chlorpyrifos and 2,4-D, respectively. Sunet al. [97] used dynamically boron hydride-reduced AgNPs as substrates and, for the first time, utilized SERS imaging technology to achieve the visual detection of pesticide residues throughout the entire process (Figure 15). This method is stable and sensitive, with an LOD of less than 1 pg/mL. It has been successfully applied to detect pesticide residues in various crops and fruit juices and to generate distribution maps of pesticide residues inside and outside fruits and vegetables.

A label-free SERS sensor was successfully developed using easily synthesized bimetallic core–shell Au@Ag nanoparticles. This SERS sensor was applied to detect acetamiprid and thiram simultaneously in apple juice and orange juice. The method showed satisfactory linearity in the range of 5–100 μM and 0.5–10 μM with limits of detection of 1.22 μM and 0.076 μM for acetamiprid and thiram in apple juice samples, respectively [98].

Xu et al. [99] developed an AChE-based SERS biosensor using Ag/Au bimetallic nanoparticles functionalized with 4-mercaptophenylboronic acid (4-MPBA) as a signal probe. The system operates through the AChE-mediated hydrolysis of ATCh in conjunction with choline oxidase (CHO), generating hydrogen peroxide that oxidizes the 4-MPBA boronic ester. This reaction decreases the Raman intensity of B–O symmetric stretching. Organophosphates (OPs) inhibit AChE activity, thereby reducing hydrogen peroxide production and subsequent SERS signal attenuation. Leveraging the Ag/Au synergistic effect, the sensor achieved an LOD of 1.7 nM for methyl parathion, demonstrating successful application in apple peel analysis. Some studies have also found that liquid SERS substrates based on Ag NPs have better signal intensity than Ag–Au NPs [100].

Flexible SERS substrates are increasingly being used due to their exceptional suitability for on-site testing. Ma et al. [101] developed a flexible SERS substrate by printing AgNPs and graphene oxide (GO) onto cellulose paper. The high-density AgNPs/GO coating enabled the ultrasensitive detection of thiram (0.26 ng/cm^2^), thiabendazole (28 ng/cm^2^), and methyl parathion (7.4 ng/cm^2^) in fruits and vegetables, demonstrating remarkable detection limits. Zhang et al. [100] developed a flexible SERS substrate by depositing monodisperse Cu–Ag nanoalloys on hexagonal boron nitride (h-BN). The h-BN/Cu–Ag nanocomposite exhibits ultrahigh SERS activity, achieving LODs of 10^−16^ M, 10^−12^ M, and 10^−9^ M for crystal violet, thiram, and ricyclazole, respectively. A simple drip-dry process enabled direct integration with polyethylene terephthalate (PET) to create low-cost flexible sensors. This copper-based platform demonstrated ultrasensitive detection of tricyclazole residues on tomato surfaces, with linear ranges of 10^−4^ to 10^−9^ M (thiram) and 10^−5^ to 10^−9^ M (tricyclazole).

With increasing awareness of environmental protection, there is growing attention being paid to renewable SERS substrates. Huang et al. [102] engineered a regenerable SERS biosensor using the interface-confined self-assembly of CdS nanowires and Ag NPs. This hierarchical nanostructure amplifies Raman signals of carbamate pesticides through electromagnetic hotspot generation, demonstrating enhancement factors of 10^5^ for metolcarb, carbaryl, and aldicarb sulfone. The substrate enables quantitative detection from 1 μg/L to 100 mg/L, with ultralow LODs of 0.81 μg/L (metolcarb), 81 ng/L (carbaryl), and 720 ng/L (aldicarb sulfone). The nanofilm’s renewable capability (>30 cycles) and 94% signal reproducibility were validated in vegetable extracts, showing less than 5% matrix interference. This plasmon–exciton coupling strategy achieves multiplex pesticide screening with smartphone-compatible spectral analysis. Liou et al. [103] developed an eco-friendly SERS platform by synergistically combining cellulose nanofibers with plasmonic silver nanoparticles (CNF–AgNPs) through a green fabrication process. This flexible nanocomposite addressed thiabendazole (TBZ) detection challenges by exploiting pH-mediated charge reversal (pH < 4.65) to overcome TBZ’s hydrophobic limitations, achieving 10^3^ signal amplification through optimized AgNP–TBZ interactions. The sensor demonstrated quantitative TBZ analysis in apples across 1–100 ppm with 5 ppm detection limits. Remarkably, the CNF matrix enhanced nanoparticle stability (>95% SERS activity retention after 8 weeks of storage) while enabling substrate recyclability through mild acid treatment.

Magnetic nanostructures also contribute to the formation of strong plasmonic hotspots and improve SERS sensor performance. Shan et al. [104] developed Ag/Fe_3_O_4_ nanocomposites through a facile one-pot synthesis, demonstrating exceptional superparamagnetic behavior. The ultra-small Fe_3_O_4_ nanoparticles act as magnetic scaffolds that assemble adjacent Ag NPs into plasmonic hotspots under magnetic guidance. This magnetically responsive architecture enables the real-time field monitoring of aquatic pesticide contamination via SERS, as well as high-sensitivity agricultural product residue analysis through magnetic concentration. The synergistic magnetic-optical properties stem from precisely controlled interparticle spacing (<5 nm) and localized surface plasmon resonance enhancement factors exceeding 10^4^.

Wei et al. [105] developed a silver-functionalized SERS substrate composed of AgNPs @ silver nanowires embedded in a polydimethylsiloxane (AgNP@AgNW/PDMS) network. The unique network structure provides abundant SERS hotspots and improves the surface’s adsorption capacity for target molecules. The prepared AgNP@AgNW/PDMS shows impressive performance with excellent reusability (10 cycles), adequate reproducibility, low LODs (10^−10^ M for thiram and 10^−11^ M for malachite green), a high refractive index, and a flexible and robust structure. It was successfully applied to detect pesticides in plants and aquatic products.

**Figure 16:**
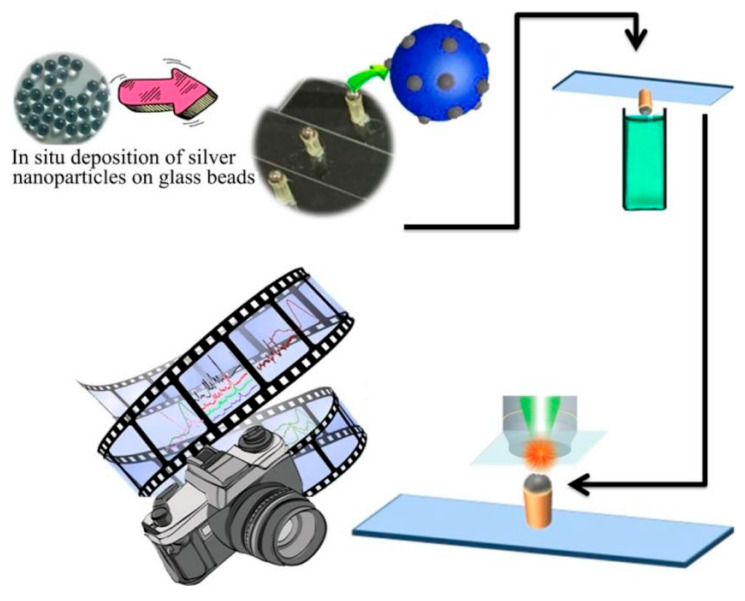
Schematic illustration of nonplanar SERS base for immersion dry measurement [106]. Adapted with permission from ref. [106]. Copyright 2019, Elsevier.

Traditional SERS substrates typically employ planar structures, which suffer from nanoparticle deposition heterogeneity and the “coffee-ring” effect caused by conventional drip-dry measure protocols. To address these limitations, Tang et al. [106] developed a nonplanar SERS substrate through AgNP deposition via Ag^+^ reduction on glass beads, utilizing a dip-dry measure approach (Figure 16). This three-dimensional architecture enables homogeneous SERS signal generation for rapid pesticide quantification in apples without requiring labeling. The optimized substrate demonstrated the quantitative detection of chlorpyrifos (linear range: 50–600 ng/mL, LOD: 10 ng/mL) and imidacloprid (linear range: 50–700 ng/mL, LOD: 50 ng/mL), with both detection limits being 100-fold below China’s maximum residue limits for apples. This methodology provides a field-deployable solution for food safety monitoring through minimized matrix interference and enhanced signal reproducibility.

Xu et al. [107] fabricated plasmonic AuNP arrays at air–water interfaces using mesoporous silica templates, creating ordered AuNP-embedded silica films (AuNPs@MSF) with sub 10 nm interparticle gaps. The confined AuNPs within silica channels exhibited enhanced electromagnetic fields, generating SERS hotspots that amplified molecular signatures 10^5^-fold. This architecture demonstrated exceptional ambient stability (>3 months, RSD = 3.1%) and suppressed nanoparticle aggregation during synthesis. The AuNPs@MSF sensor achieved ultrasensitive 2,4-D detection (LOD: 0.79 pg/mL) across four orders of magnitude (10^−2^–10^3^ ng/mL), outperforming label-free methods. Extended pesticide screening yielded sub-pg/mL LODs for pymetrozine (1.04 pg/mL) and thiamethoxam (1.21 pg/mL) within 0.1–1000 ng/mL linear ranges, proving effective in food and environmental matrices.

**Figure 17:**
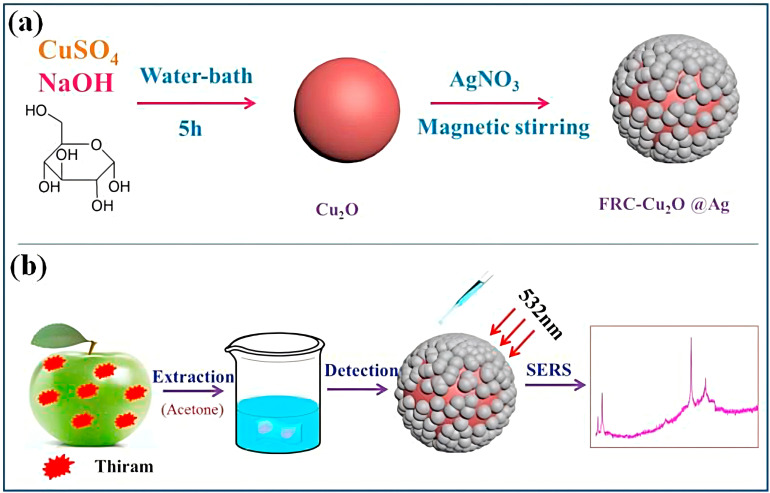
Schematic illustration of the formation of FRc-Cu,O@Ag microsphere (**a**) and the formed substrate used to detect thiram on the apple peel (**b**) [108]. Adapted with permission from ref. [108]. Copyright 2021, Elsevier.

Yao et al. [108] enhanced copper-based biosensor sensitivity through integrated sample pretreatment, developing Ferrero Rocher-inspired Cu_2_O@Ag microspheres (FRC-Cu_2_O@Ag) via in situ redox synthesis (Figure 17). This SERS substrate demonstrated significant signal enhancement upon adsorption of 4-mercaptobenzoic acid and thiram, achieving ultrasensitive detection through synergistic electromagnetic and chemical enhancement mechanisms. The sensor detects dithiocarbamoyl residue on apple peels with an LOD of 1.2 × 10^−7^ M.

The advantages of SERS include (1) ultrahigh sensitivity (with single-molecule resolution in optimized systems), (2) high selectivity, (3) robust reproducibility, and (4) non-destructive operation. The challenges of SERS include (1) substrate-dependent signal-to-noise (SNR) ratio, (2) technical complexity (requires precise synthesis of monodisperse metallic nanostructures), (3) matrix interference, and (4) analyte limitation (restricted to molecules with surface-binding functional groups or π-conjugated systems). Future directions of SERS are (1) engineering hybrid substrates combining metallic nanostructures with 2D materials (graphene, MXenes) to enhance stability, (2) implementing roll-to-roll fabrication methods for low-cost flexible SERS platforms, and (3) developing machine learning-assisted spectral deconvolution algorithms for low-SNR environments.

#### 3.2.4. Other Optical Nanosensors

Other types of light, such as visible and ultraviolet light, have also been exploited for optical sensing applications. Faghiri et al. [109] developed a dual-functional biosensor combining magnetic solid-phase extraction (MSPE) with Cu@Ag-nanoparticle-based colorimetric detection for malathion analysis in agricultural and environmental samples. The system utilizes Fe_3_O_4_/graphene oxide (GO) nanocomposites as MSPE adsorbents for analyte pre-concentration. Upon exposure to malathion, Ag–S covalent bonds form between the pesticide and Cu@Ag nanoparticles, inducing nanoparticle aggregation. This results in a visible color change from a brass-colored dispersion to purple-gray aggregates, detectable both by eye and UV–vis spectroscopy, with a λ_max_ shift from 420 nm to 550 nm. This integrated approach achieved a limit of detection (LOD) of 14 μg/L.

He et al. [110] developed a simple and sensitive chemiluminescence (CL) sensor array based on luminol-functionalized AgNPs for the discriminative detection of organophosphorus and carbamate pesticides. By monitoring three intrinsic CL response parameters—intensity, time-to-onset, and time-to-peak—this system generated pesticide-specific luminescent fingerprints. Pattern recognition via principal component analysis (PCA) enabled clear discrimination among five pesticides (dichlorvos, trichlorfon, carbaryl, chlorpyrifos, and carbofuran) at 24 μg/mL. Validation with 20 blind samples demonstrated 95% identification accuracy through cross-validation, confirming the array’s capability for rapid pesticide screening without requiring complex instrumentation.

Bala et al. [48] developed a straightforward biosensor for the rapid detection of methyl phosphate, a highly toxic organophosphate pesticide. The system employs pesticide-specific aptamers as recognition elements and AuNPs as optical indicators. In the absence of pesticide, aptamers maintain a random coil conformation that stabilizes dispersed AuNPs, rendering the solution red. Upon target pesticide binding, aptamer structural rigidity increases, triggering AuNP aggregation and a visible color shift from red to blue. This biosensor demonstrated a detection range from 0.01 nM to 1.3 μM with an ultralow LOD of 0.01 nM.

Shrivas et al. [111] developed a smartphone-integrated paper sensor utilizing Cu@Ag core–shell nanoparticles for phenthoate detection in water and food samples. The sensing mechanism relies on phenthoate’s selective binding to AgNPs on the Cu core, inducing visible color changes on the paper substrate. This portable system achieved an LOD of 15 μg/L, offering a cost-effective and user-friendly alternative to conventional analytical instrumentation.

Kamyabi et al. [112] fabricated an enzyme-free electrochemiluminescence (ECL) platform using a CuS/carbon quantum dots (CQDs)/graphitic carbon nitride (g-C_3_N_4_) nanocomposite. The system employs boron nitride quantum dots (BNQDs) as co-reactants in the cathodic potential region, enabling operation at negative potentials. The nanocomposite, comprising g-C_3_N_4_ nanosheets supporting CQDs and CuS nanoparticles, was uniformly deposited on glassy carbon electrodes. This ternary structure demonstrates synergistic electro-optic enhancement, with BNQDs boosting photoelectric signals 3.8-fold compared to individual components. The platform achieves a linear detection range of 1.0 × 10^−15^ to 5.0 × 10^−9^ M for diazinon with an LOD of 2.2 × 10^−16^ M. The amplified ECL signal arises from efficient electron transfer among the nanocomposite components and BNQD-mediated signal amplification.

Table 4 summarizes the representative studies of optical nanosensors for pesticide detection in agricultural products.

### 3.3. Other Biosensors

#### 3.3.1. Terahertz Spectral Biosensor

Terahertz (THz) waves are electromagnetic waves with frequencies ranging from 0.1 THz to 10 THz (wavelengths from 0.03 to 3 mm). Historically, the “THz gap”—limited by incoherent thermal sources and cryogenic bolometers—restricted practical applications. Recent breakthroughs in quantum cascade lasers and photoconductive antennas have enabled tabletop THz time-domain spectroscopy (THz-TDS) systems with bandwidths exceeding 3 THz [114]. THz biosensing exploits dielectric-sensitive resonance shifts in subwavelength structures (such as metasurfaces and graphene plasmonics) through local field enhancement via spoof surface plasmon polaritons and resonance frequency modulation [115]. Terahertz spectroscopy offers highly sensitive molecular detection capabilities. The development and testing of flexible, compact, and highly sensitive THz sensors have been a hot research topic in recent years. However, persistent limitations include fabrication complexity and decreased sensitivity caused by analyte–structure decoupling.

Xu et al. [116] prepared a non-metamaterial flexible graphene-based terahertz biosensor. Pesticides strongly interact with the π electrons in graphene, shifting the Fermi level, altering terahertz wave absorption, and, thus, enabling detection. The authors constructed a flexible device using gold or conductive tape as a back-reflector and detected pesticide molecules on the bio-interface surface. This sensor successfully detected chlorpyrifos methyl in apple peels with an LOD of 0.13 mg/L over the concentration range of 0.02–50 mg/mL. The sensor demonstrated stability and robustness over 1000 bending cycles.

Lang et al. [117] fabricated a single-layer graphene metamaterial sensor operating in the 0–1 THz frequency range, achieving high-sensitivity detection characterized by spectral amplitude changes. Phosphonates can alter graphene’s Fermi level through π–π stacking, facilitating their detection. The sensor demonstrated an LOD of 0.01 μg/mL within a linear range of 0–2 μg/mL and showed promising potential for agricultural product detection.

Wang et al. [118] constructed subwavelength periodic micro carbon nanotube structures on silicon substrates, leveraging the excellent conductivity of carbon nanotubes. When THz waves are incident, surface plasmon polariton (SPP) resonance occurs at the interface between the carbon nanotube film and silicon, exposing the sensor to the analyte. The sensor’s LOD for 2,4-D and chlorpyrifos were 1.38 × 10^−2^ ppm and 2.0 × 10^−3^ ppm, respectively.

#### 3.3.2. Lateral Flow Assay (LFA)

Lateral flow assay (LFA) utilizes capillary action to drive sample migration along a test strip, achieving target detection through antigen–antibody-specific recognition or nucleic acid hybridization. LFA enables the qualitative or semi-quantitative detection of pesticide residues in approximately 20 min. Advantages include rapid on-site testing, low cost, ease of operation, and good stability.

Wang et al. fabricated a competitive fluorescent LFA using silica-core multilayered quantum dot nanobeads (SiO_2_-MQB) as fluorescent tags. This assay showed improved stability, dispersibility, and luminescence compared to traditional fluorescent beads, significantly enhancing pesticide detection sensitivity. It enabled the stable and accurate detection of imidacloprid and carbendazim within 12 min, with detection limits as low as 1.94 pg/mL and 14.79 pg/mL, respectively. The method was successfully applied to real food samples including corn, apple, cucumber, and cabbage, demonstrating its promise as a low-cost on-site monitoring tool for trace pesticide residues [119].

Zhai et al. developed an LFA strip incorporating Fe_3_O_4_ nanoparticles and metal–organic frameworks capable of detecting carbofuran in the range of 0.25–50 ng/mL with an LOD of 0.15 ng/mL, enabling both colorimetric and catalytic analysis [120].

## 4. Conclusions and Future Perspectives

Pesticide residues in agricultural products have become an increasingly significant concern in recent years. Numerous methods for detecting pesticides in agricultural products have been developed. In this review, we primarily focused on commonly used optical and electrochemical strategies that have been innovatively designed and successfully applied for pesticide detection, with particular emphasis on recognition elements such as enzymes, antibodies, aptamers, and molecularly imprinted polymers (MIPs). The working principles, advantages, limitations, and detection performance of these biosensors were systematically evaluated. These biosensors exhibit desirable characteristics including high sensitivity, portability, reliability, and cost-effectiveness, effectively addressing the drawbacks of traditional detection methods such as time-consuming sample pretreatment, the requirement for skilled personnel, and the inability to achieve on-site and real-time monitoring.

In recent years, the continuous advancement of biosensing techniques—including electrochemical methods, potentiometry, amperometry, calorimetry, optical biosensors, and immunosensors—and their associated manufacturing technologies has paved the way for the development of nanomaterial-based nanosensors with superior detection capabilities and miniaturized formats. Among these, optical biosensors, especially those based on fluorescence, surface-enhanced Raman scattering (SERS), and surface plasmon resonance (SPR), are the most mature, offering remarkable sensitivity. However, the widespread use of nanomaterials raises concerns regarding their potential environmental and biological impacts, including possible effects on human health. Therefore, a cautious approach is necessary, accompanied by thorough evaluations of their safety and environmental footprint.

Looking forward, ongoing advancements in novel materials and sensing technologies are expected to drive the continued evolution of biosensors that leverage nanomaterials and nanostructures. Next-generation nanobiosensors will deliver unprecedented sensitivity and specificity, featuring ultralow detection limits and compact sizes that enable real-time in-field detection. There remains significant demand for fluorescence-based nanosensors, such as those employing carbon dot architectures, to tackle critical applications including agricultural product analysis, environmental toxin surveillance, early disease diagnostics, and public security. Emerging platforms like smartphone-integrated biosensors—with open-architecture designs, portable paper- or barcode-based formats, renewable interfaces, user-friendly operation, intelligent data processing, and flexible substrates for SERS detection—show exceptional promise for on-site monitoring across diverse sectors.

Finally, agricultural monitoring currently faces significant challenges due to the vast diversity of pesticides used. While agricultural produce may contain numerous pesticide types, most detection methods are limited to identifying single or a few pesticide variants. Future progress will depend on developing innovative biosensor chips that integrate nanomaterials with multiplexing technologies, enabling the simultaneous screening of multiple pesticide residues through advanced detection platforms.

## Figures and Tables

**Table 2:** Representative nanosensor-related studies using enzymes as receptors for pesticide detection in agricultural products.

Enzyme Types	Detection Methods	Target Pesticides	Ref.
AChE	Fluorimetry	OPs (chlorpyrifos)	[20,25,26]
AChE	Fluorimetry	Carbamate (carbaryl)	[27]
AChE	Colorimetry	OPs	[21,22]
AChE	SERS	OPs	[28]
AChE	Amperometric	OPs (chlorpyrifos)	[29,30]
AChE	DPV	OPs (chlorpyrifos)	[31,32,33,34]
AChE	Potentiometric	Carbamate (carbaryl)	[35]
Urease	Potentiometric	OPs (glyphosate)	[36]
Butyrylcholinesterase	Photoelectrochemical	OPs	[37]
AChE	CV	Carbamate pesticides	[38]
AChE	SWV	OPs (malathion)	[39]
AChE	Relative resistance change	Methamidophos	[40]

Note: AChE, acetylcholinesterase; SERS, surface enhanced Raman spectroscopy; DPV, differential pulse voltammetry; CV, cyclic voltammetry; SWV, square wave voltammetry; OPs, organophosphate pesticides.

**Table 3:** Representative studies of electrochemical nanosensors for pesticide detection in agricultural products.

Detection Method	Nanomaterial	Targeted Analyst	Stability	Linear Range	LOD	Sample	Ref.
DPV	MnO_2_-GNPs	Carbaryl; fenobucarb; carbosulfan	/	Carbaryl, 1–30 μM; Fenobucarb, 5–80 μM; Carbosulfan, 50–400 μM	Carbaryl, 0.30 μM; Fenobucarb, 1.40 μM; Carbosulfan, 15.15 μM	Rice and rice-field water	[72]
Potentiometric	Urease/Au NPs	Glyphosate	180 days	0.5–50 ppm	0.5 ppm	Tap waters	[36]
Amperometric	Nafion/AChE-cSWCNT/MWCNT/AuNPs-Au	Methyl parathion; monocrotophos; chlorpyrifos; endosulfan	2 months	0.1–130 µM	Methyl parathion, 1.9 nM; monocrotophos, 2.3 nM; chlorpyrifos, 2.2 nM; endosulfan, 2.5 nM	Cabbage, Onion, Spinach	[30]
Amperometric	Ag NPs–CGR–NF/GCE	Chlorpyrifos and carbaryl	30 days	Chlorpyrifos, 1.0 × 10^−13^–1 × 10^−8^ M; carbaryl, 1.0 × 10^−12^–1 × 10^−8^ M	Chlorpyrifos, 5.3 × 10^−14^ M; carbaryl, 5.45 × 10^−13^ M	Tap water, lake water sample	[33]
DPV	AChE-Chit/Pd@Au NWsN/GCE	Malathion	/	0.1 pM–100 nM	0.037 pM	Tap water	[73]
CV	PVA-AWP/Fe–Ni NP/AChE	Phosme	>30 days	1 × 10^−10^–5 × 10^−9^ M	0.1 nM	Olive oil	[69]
CV	Nano Ag-nano Fe_3_O_4_	Methomyl	/	2.97 × 10^−5^–3.47 × 10^−4^ mol/L	2.08 × 10^−5^ mol/L	Vegetable	[74]
CV and DPV	Au@MWCNTs/GCE	Dichlorvos	/	1–120 μM	5 nM	Leaf lettuce	[60]
SWV	Zr-BDC-rGO	Methyl parathion	28 days	0.001–3.0 μg/mL	0.5 ng/mL	Chinese cabbage	[70]
SWV	AChE-CS/3DG-CuO NFs/GCE	Malathion	20 days	3 pM–46.67 nM	0.92 pM	River and lake water	[39]
DPV	MXene-rGO/Ed-Ab/FTO	Endosulfan	21 days	0.1–1 ppt	0.497 ppt	Water, leaf and root extracts	[45]
CV and DPV	SPCE|CNTs/ZrO_2_/PB/Nf|GMP-AChE	Organophosphorus		1.0 × 10^−3^–10 ng/mL	5.6 × 10^−4^ ng/mL	Chinese cabbage	[24]
CV	AuNPs/MPS/Au	Carbamate	28 days	0.003–2.00 μM	1 nM	Fruits	[38]
DPV	AChE/MHCS/GCE; AChE/Fe_3_O_4_@MHCS/GCE	Malathion	30 days	0.01–600 ppb	0.0182 ppb	Pear	[31]

Note: LOD, limits of detection; DPV, differential pulse voltammetry; GNPs, graphene nanoplateletss; AChE, acetylcholinesterase; MWCNTs, multi-walled carbon nanotubes; cSWCNT, carboxylated single-walled carbon nanotubes; GCE, glassy carbon electrode; CGR, carboxymethyl graphene; CV, cyclic voltammetry; CS, chitosan; NWsN, nanowires network; PVA, polyvinyl alcohol; AWP, azide-unit water-pendant; rGO, reduced graphene oxides; BDC, Benzo-1,4-dicarboxylic acid; 3DG, three-dimensional graphene; FTO, fluorine-doped tin oxide; PB, Prussian Blue; Ed, endosulfan; Ab, antibody; MPS, 3-mercaptopropyl-trimethoxysilane; MHCS, mesoporous hollow carbon spheres.

**Table 4:** Representative studies of optical nanosensors for pesticide detection in agricultural products.

Detection Method	Nanomaterial	Targeted Analyst	Linear Range	LOD	Sample	Ref.
Fluorimetry	6-carboxy-fluorescein labeling aptamer MNPs	Trichlorfon, glyphosate, and malathion	0.0001 –10 mg/L	Trichlorfon, 72.20 ng/L; glyphosate, 88.80 ng/L; malathion 195.37 ng/L	Lettuce, carrot	[47]
SERS	Ag NPs coated glass bead	Chlorpyrifos, imidacloprid	Chlorpyrifos, 50–600 ng/mL; Imidacloprid, 50–700 ng/mL	Chlorpyrifos (10 ng/mL); imidacloprid (50 ng/mL)	Apple	[106]
SERS	AuNPs deposited in mesoporous silica film	2,4-D, pymetrozine, and thiamethoxam	2,4-D, 10^−2^–10^−3^ ng/mL; pymetrozine, 0.1–1000 ng/mL; thiamethoxam, 0.1–1000 ng/mL	2,4-D (0.79 pg/mL), pymetrozine (1.04 pg/mL); thiamethoxam (1.21 pg/mL)	Tap water, apple, and milk	[107]
SERS	Cellulose nanofibers coated with AgNPs	Thiabendazole	1–100 ppm	5 ppm	Apple	[103]
Fluorimetry	Rhodamine B-modified Ag@Au NPs	Organophosphorus pesticides	0.0033–0.28 ng/mL	0.0018 ng/mL	Lake water, tomato, and apple	[80]
SERS	Au@Ag nanoparticles	Acetamiprid, thiram	Macetamiprid, 5–100 μM; thiram, 0.5–10 μM	Acetamiprid (1.22 μM), thiram (0.076 μM)	Apple juice	[98]
SERS	4-mercaptophenylboronic acid modified Ag/Au bimetallic nanoprobes	Parathion-methyl	5 × 10^−9^–5 × 10^−4^ M	1.7 nM	Apple	[99]
SERS	Au-Ag colloid nanoparticles	Deltamethrin	0.01–500 ppm	0.01 ppm	Tea	[113]
Colorimetry	Citrate capped Cu@Ag core–shell nanoparticles	Phenthoate	50–1500 μg/L	15 μg/L	Water and food	[111]
SERS	Hexagonal boron nitride coated with highly dense and monodisperse Cu-Ag nanoalloys	Thiram and tricyclazole	Thiram, 10^−4^–10^−9^ M; tricyclazole, 10^−5^–10^−9^ M	Thiram (1 pM) and tricyclazole (1 nM)	Tomato	[100]
SERS	Ferrero^®^ chocolate-like Cu_2_O@Ag microspheres	Thiram	1.2 × 10^−7^–2.4 × 10^−6^ M	1.2 × 10^−7^ M	Apple	[108]
SERS	SPE modified by dropping Ag NPs	Acetamiprids	0.1–1000 μM	0.04 μM	*Brassica chinensis* L.	[67]
Fluorimetry	CDs/AChE/GO	Chlorpyrifos	1–25 ppb	0.14 ppb	Tap water	[25]
Fluorimetry	CDs-AChE aerosol	Paraoxon; parathion; dichlorvos; deltamethrin	10^−5^–10^−12^ M	Paraoxon, 1.2 pM; parathion, 0.94 pM; dichlorvos. 11.7 pM; deltamethrin, 0.38 pM	Apple	[20]
Fluorimetry	AuNP/Ab/ssDNAs; MNP-OVA; Au@Pt/ssDNAs	Parathion, triazophos, and chlorpyrifos	0.01–31.62 ng/mL	Parathion, 9.88 ng/kg; triazophos, 3.91 ng/kg; chlorpyrifos, 1.47 ng/kg	Cabbage, apple, pear, and rice	[43]
Colorimetry	Fe_3_O_4_/GO	Malathion	0.001–5 mg/L	0.014 mg/L	Apple and river water	[109]
Fluorimetry	CQDs	viz., quinalphos 25 EC; thiamethoxam 25 WG; propargite 57 EC	0.2–5000 ng/mL	viz., quinalphos 25 EC, 0.2 ng/mL; thiamethoxam 25 WG, 1 ng/m; propargite 57 EC, 10 ng/mL	Tea	[82]
Fluorimetry	CdSe/ZnS/QD/Ab	Triazophos	10–25 μg/L	0.508 ng/L	Apple, pear, cucumber, and rice	[78]
Fluorimetry and Colorimetry	B, N-doped CQDs	Carbaryl	0.20–150 μg/L	0.06 μg/L	Lake water and tap water	[27]
Fluorimetry	OPCD@UiO-66-NH_2_	Quinalphos	0–16 μM	0.3 nM	Tomato and rice	[86]
Fluorimetry	Red-emission carbon dots	Pyrethroid	1–120 μg/L	0.89 μg/L	Tea and grapes	[88]
SPR	Poly nanofilms composed of N-methacryloyl-L-cysteine methyl ester, ethylene glycol dimethacrylate, 2-hydroxyethy methacrylate	Coumaphos	0.1–250 ppb	0.001 ppb	Honey	[53]
SERS	Silver colloid	Chlorpyrifos; 2,4-D	0.001–1000 mg/L	Chlorpyrifos, 1.28 × 10^−9^ M; 2,4-D, 2.47 × 10^−10^ M	Apple	[96]

Note: SPR, surface plasmon resonance; SERS, surface enhancement of Raman scattering; LOD, limit of detection; SPE, screen-printed electrode; MNPs, magnetic nanoparticles complex; GO, graphene oxide; CQDs, carbon quantum dots; ssDNAs, single-stranded DNA; OPCD, carbon dot derived from orthophenylenediamine; OVA, ovalbumin.

## Data Availability

No new data were created or analyzed in this study. Data sharing does not apply to this article.
